# In silico identification and comparative analysis of differentially expressed genes in human and mouse tissues

**DOI:** 10.1186/1471-2164-7-86

**Published:** 2006-04-21

**Authors:** Sheng-Ying Pao, Win-Li Lin, Ming-Jing Hwang

**Affiliations:** 1 Institute of Biomedical Engineering, National Taiwan University, Taipei, Taiwan; 2 Institute of Biomedical Sciences, Academia Sinica, Taipei, Taiwan

## Abstract

**Background:**

Screening for differentially expressed genes on the genomic scale and comparative analysis of the expression profiles of orthologous genes between species to study gene function and regulation are becoming increasingly feasible. Expressed sequence tags (ESTs) are an excellent source of data for such studies using bioinformatic approaches because of the rich libraries and tremendous amount of data now available in the public domain. However, any large-scale EST-based bioinformatics analysis must deal with the heterogeneous, and often ambiguous, tissue and organ terms used to describe EST libraries.

**Results:**

To deal with the issue of tissue source, in this work, we carefully screened and organized more than 8 million human and mouse ESTs into 157 human and 108 mouse tissue/organ categories, to which we applied an established statistic test using different thresholds of the *p *value to identify genes differentially expressed in different tissues. Further analysis of the tissue distribution and level of expression of human and mouse orthologous genes showed that tissue-specific orthologs tended to have more similar expression patterns than those lacking significant tissue specificity. On the other hand, a number of orthologs were found to have significant disparity in their expression profiles, hinting at novel functions, divergent regulation, or new ortholog relationships.

**Conclusion:**

Comprehensive statistics on the tissue-specific expression of human and mouse genes were obtained in this very large-scale, EST-based analysis. These statistical results have been organized into a database, freely accessible at our website , for easy searching of human and mouse tissue-specific genes and for investigating gene expression profiles in the context of comparative genomics. Comparative analysis showed that, although highly tissue-specific genes tend to exhibit similar expression profiles in human and mouse, there are significant exceptions, indicating that orthologous genes, while sharing basic genomic properties, could result in distinct phenotypes.

## Background

High-throughput analysis of gene expression offers a powerful means of studying how genes work and of uncovering the secrets encoded in genome sequences. Differential gene expression, which plays a key role in various cellular processes, can be quantified by analyzing a large number of transcription products. To do so, several large-scale transcript detection technologies have been developed, chief among which are variants of microarray technology [1,2], expressed sequence tags (ESTs) [3], and serial analysis of gene expression (SAGE) [4]. Although each of these has its own limitations [5-10], combined with bioinformatics and statistical analysis, they have been successful in revealing genes expressed differentially in different tissues or in different physiological or phenotypical states and in yielding unprecedented insights into the complicated interactions of expressed genes and their cellular functions [10-12].

In this work, the EST database for human and mouse was analyzed to identify tissue-specific and differentially expressed genes. ESTs are "single-pass" sequences of randomly selected clones of expressed genes from specific tissues, organs, or cell types [3]. Because EST clone frequency is, in principle, proportional to the expression level of its corresponding gene in the sampled tissue, tissue-specific or differentially expressed genes can be identified by their significantly different number of EST transcripts seen in unbiased cDNA libraries from different tissues [13,14]. Data on ESTs have been accumulating in the public domain for more than a decade and, at the present time, there are more than 5.3 million entries for human and more than 3.9 million for mouse. ESTs are also well-organized in UniGene clusters, which are linked to other types of information [15], allowing gene-centered analysis.

Several EST-based tools have been developed to extract gene expression profiles. BodyMap [16] uses its own standardized and non-normalized EST libraries exclusively for high-quality expression profiling, but its sample size of less than half a million EST sequences from 64 human and 39 mouse tissues may not give a complete picture of genome-wide gene expression [17,18]. TissueInfo [17] and ExQuest (Expressional Quantification of ESTs) [18] are similar to each other in that they both compare EST sequences against dbEST [19] using MegaBlast [20] to extract the tissue information associated with each matching EST. However, they do not provide quantified expression profiles for genes identified as differentially expressed under a specified statistical cut-off.

The present work adopted a gene-centered strategy, taking advantage of the well-annotated and widely used UniGene clusters [15], in which ESTs are grouped in units of genes. This allows searching of genes, eliminates the need for sequence comparison, a computationally expensive procedure given the number of ESTs accumulated in the database, and avoids difficulties in matching and distinguishing between homologous genes.

Because some of the EST libraries were derived from unspecified tissues or under artificially modified expression conditions, we removed 1,898 such human libraries (out of 8,145; 23.3%) and 211 such mouse libraries (out of 841; 25.1%) from our analysis (see Methods) and organized the rest into a hierarchy of manually curated tissue/organ classes. These EST data were then subjected to the statistical test of Audic and Claverie [21], known as the A-C test, which has been shown to perform better than several other statistical tests for pairwise comparison of gene expression data in tag sampling experiments [22]. In all, genes preferentially expressed in different tissues at various levels of specificity in 157 human and 108 mouse tissues were identified. The results were evaluated by comparison with microarray results for 17 tissues [23] and with the reported expression of several genes in different tissues and the genes reported to be expressed in a given tissue [24-29]. The expression profiles of human-mouse orthologous genes that were differentially expressed in normal tissues were also compared and analyzed.

## Results

### Identification of differentially expressed genes

**Figure 1:**
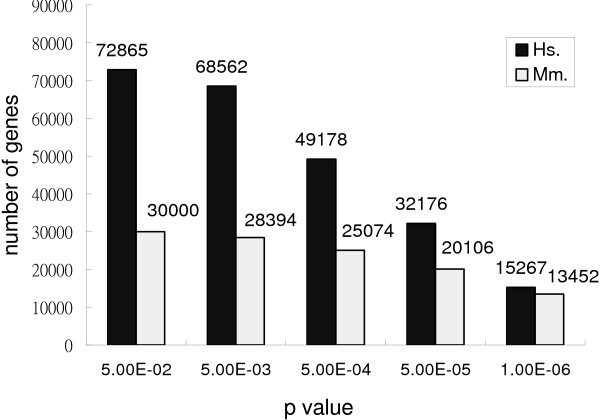
**Number of genes identified as differentially expressed using different *p *value thresholds in 94 human and 99 mouse normal tissues**.

We used the A-C statistical test to identify differentially expressed genes in 94 normal human and 99 normal mouse tissues (see website) using *p *value thresholds of 5E-2, 5E-3, 5E-4, 5E-5, and 1E-6, which are used hereafter to measure the extent of differential expression. As expected, the number of differentially expressed genes decreased as we lowered the *p *value threshold (Fig. 1). Table 1 further shows that most genes were expressed in only a few tissues: at *p *< 1E-6, ~90% of human genes and ~85% of mouse genes were expressed in ≤ 3 tissues.

### Comparison with microarray and published results

**Figure 2:**
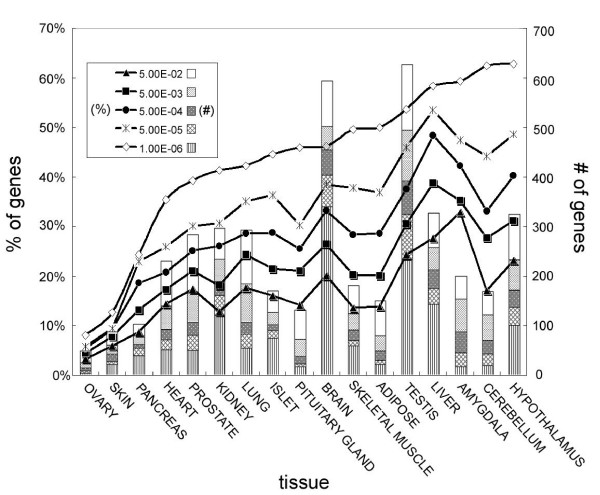
**Comparison of human genes identified as differentially expressed by EST analysis with the microarray data for 17 tissues**. The percentages of genes identified using different *p *values that were found to be up-regulated by 3-fold in a microarray from GNF SymAtlas [32] are indicated by the lines and the number (#) of genes by the bars.

To evaluate our results, we compared the human genes identified as differentially expressed using different *p *values with the microarray data provided by Su et al. [23] for 17 tissues (Fig. 2). For the comparison, we first identified, for each tissue, differentially expressed genes under a specified *p *value cut-off, and extracted those that were also included in the microarray experiments. Of these extracted genes, we identified those showing over-expression (i.e., 3-fold higher than the median expression) in the microarray experiment. We then calculated the percentage of genes identified as differentially expressed under various p-value thresholds in our data that were also over-expressed in the microarray experiment. The results showed that, for all 17 tissues, this percentage increased as more genes without significant tissue specificity were filtered out, i.e. as the *p *value threshold was set lower. Furthermore, at a threshold of 1E-6, this percentage varied significantly across tissues, ranging from less than 10% for ovary and skin to ~60% for liver and three brain tissues. These results are consistent with an earlier analysis showing that the correlation between microarray and EST data for genes differentially expressed in brain (r^2 ^= 0.43) is much higher than that for genes differentially expressed in the pancreas (r^2 ^= 0.02) or ovary (r^2 ^= 0.03) [24].

To further evaluate the usefulness of our work, we compared our results with published data for several known tissue-specific genes. *KLK3*, *TMEM10*, and *AMBP *are three notable examples. *KLK3*, a member of the kallikrein gene family, is prostate-specific [25]. In our analysis, *KLK3 *was identified in the prostate with a very high specificity (*p *< 1E-99). *TMEM10*, a recently reported novel human brain-specific gene [26], was also found to be specifically expressed in the forebrain (*p *= 2.57E-27), whole brain (*p=*9.49E-20), hippocampus (*p *= 8.77E-10), and hypothalamus (*p *= 2.43E-08). Alpha-1-microglobulin/bikunin precursor°(*AMBP*) is a well known gene exclusively expressed in liver both in human and mouse [27,28], and our data showed that *AMBP *was expressed with very high specificity in the liver (*p *< 1E-99) for both human and mouse. In addition to these three specific examples, 97.2% of the human placenta-specific genes identified by Miner and Rajkovic [29] and all of the human brain-specific genes reported by Huminiecki et al. [24] were found to show the same tissue specificity (*p *< 1E-6) in our study (data not shown).

### Correlation analysis of human and mouse orthologous genes

Of the 10,307 human and mouse orthologous gene pairs downloaded from the NCBI HomoloGene database, 7,853 contained sufficient EST data to qualify for the A-C test; 1,268 of these were expressed in fewer than 3 tissues and were therefore excluded from the *p *value correlation analysis, as described in the Methods. For the remaining 6585 gene pairs, the average *p *value correlation coefficient was only 0.20 (Table 2). Of the 6585 gene pairs, we further extracted genes differentially expressed, as defined by a given *p *value threshold, in at least one tissue in human and also one tissue in mouse. That is, for example, when the threshold was set at 1E-6, genes expressed in at least one human tissue and one mouse tissue with *p *< 1E-6 were included in the correlation analysis, even though their expression in other tissues might not meet the threshold. As can be seen from Table 2, as the threshold for defining tissue-specific orthologs was lowered, the correlation became better, and orthologs differentially expressed in at least one tissue with *p *< 1E-6 in human and mouse respectively had a strong correlation (0.6).

**Figure 3:**
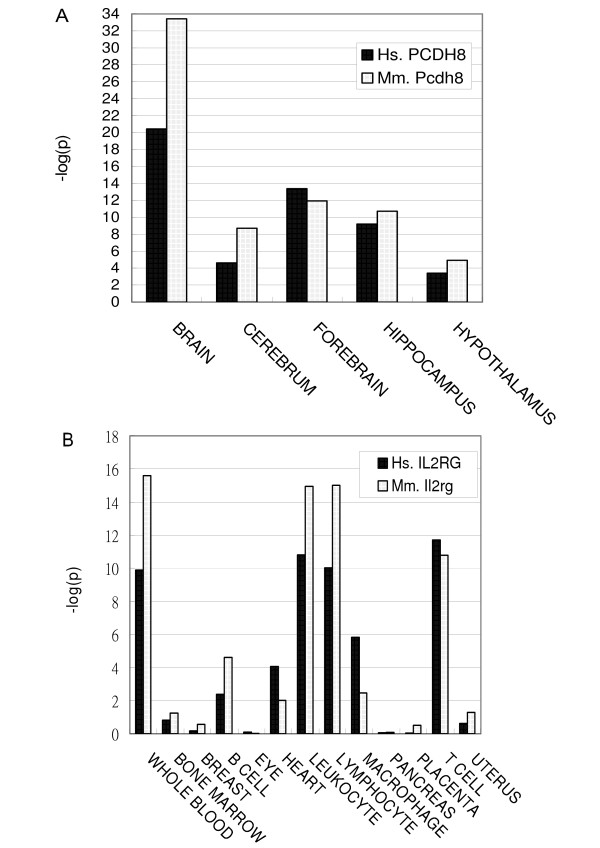
**Tissue expression profiles of strongly positively correlated orthologs**. (A) *PCDH8 *and *Pcdh8 *(p value correlation r = 0.92), (B) *IL2RG *and *Il2rg *(r = 0.9). Tissue specificity is shown as -log(*p*).

The correlation coefficient measures the extent to which the human and mouse orthologous genes show the same tissue specificity. We further dissected the strength of this association for those orthologs with significant tissue specificity, *i.e*., those expressed in at least one tissue with *p *< 1E-6. As shown in Table 3, the results demonstrated that 40% of the qualified gene pairs showed a very high positive correlation (r ≥ 0.8), indicating that these orthologs exhibited very similar expression patterns in human and mouse. The pair showing the strongest correlation (r = 0.92) was human *PCDH8 *and its mouse counterpart, *Pcdh8*, both of which were found to be expressed in cerebrum, forebrain, hypothalamus, hippocampus, and whole brain (Fig. 3A). This result is in agreement with previous experimental findings that this gene is expressed predominantly in the brain in both human and mouse [30].

Another example is *IL2RG*, which is reported to be essential for the development of T and NK lymphocytes and mutation of which can cause severe combined immunodeficiency disorder (SCID) [31]. We found that human *IL2RG *and its mouse ortholog *Il2rg *were both expressed in 13 tissues with highly similar tissue specificities (r = 0.9), and, in accordance with their function [31], were preferentially expressed in T cells, lymphocytes, leukocytes, and whole blood (Fig. 3B).

**Figure 4:**
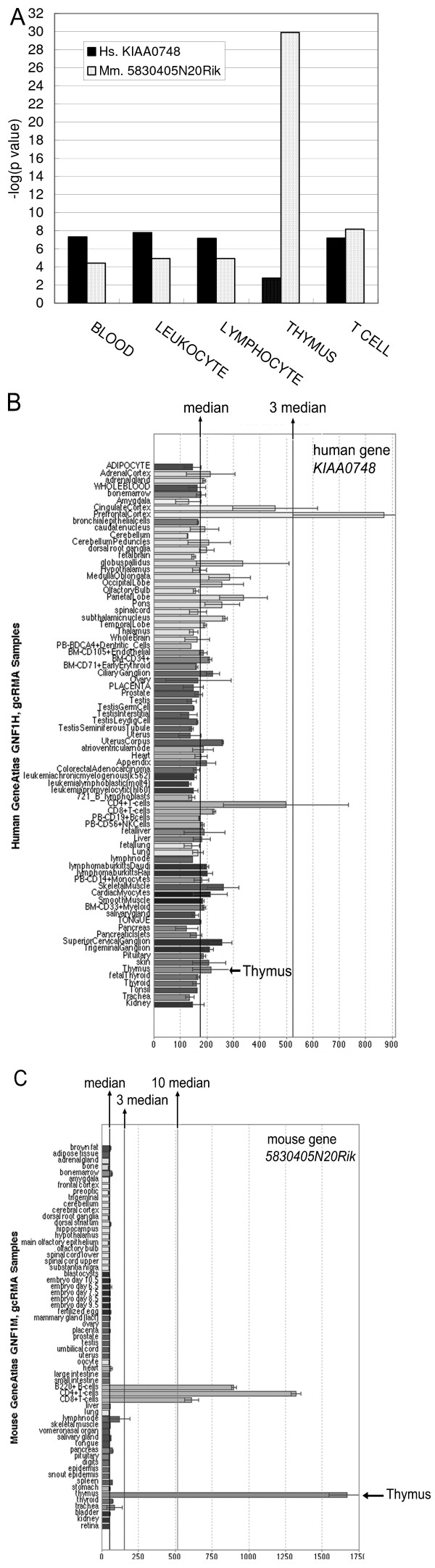
**Tissue expression profiles of human *KIAA0748 *and its designated mouse ortholog, *5830405N20R*ik**. (A) EST analysis of the present work (this pair showed the strongest negative correlation of r = -0.99, which was a consequence of their divergent level of expression in the thymus). (B) and (C) microarray data for mouse (B) and human (C) [32], which also showed this discrepancy. In mouse, the expression of this gene in the thymus was more than 10 times the median value, while, in humans, it was around the median, much lower than the value of 3 times the median, the level generally used as the threshold for preferential expression. (B) and (C) were exported from GNF SymAtlas [32].

**Figure 5:**
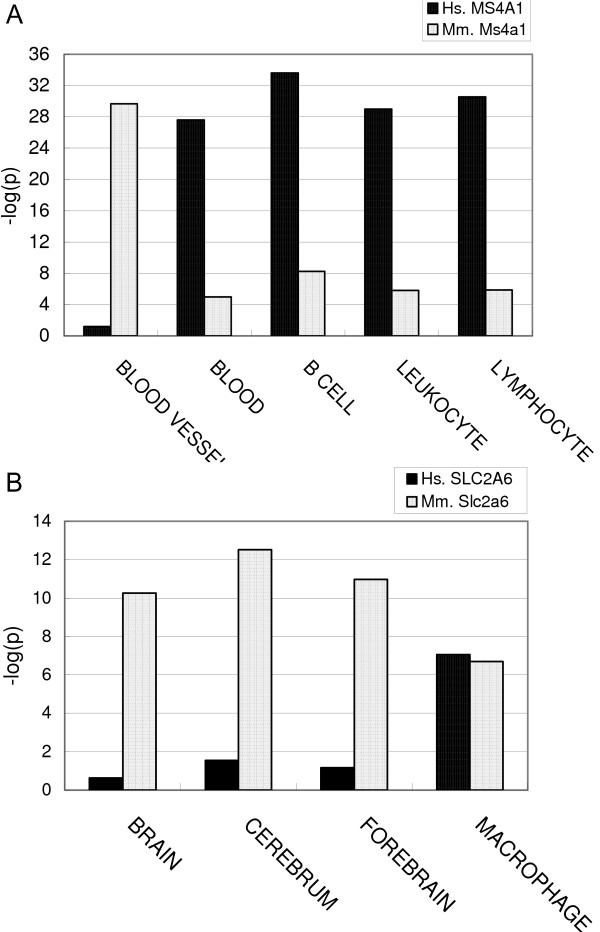
**Tissue expression profiles of strongly negatively correlated orthologs**. Tissue expression profiles of (A) human *MS4A1 *and its mouse ortholog *Ms4a1*, showing that the main discrepancy is in the blood vessel (r = -0.96), and (B) human *SLC2A6 *and its mouse ortholog *Slc2a6*, showing that the main discrepancy is in three brain tissues (r = -0.87).

In contrast, very strong negative correlations of orthologous genes (r ≤ -0.8), indicative of different tissue specificities, were found in three pairs (Table 3). The strongest negatively correlated pair (r = -0.99) was human *KIAA0748 *and its mouse counterpart, *5830405N20Rik*. As shown in Fig. 4A, they were both expressed in blood, leukocytes, lymphocytes, thymus, and T cells, but a large discrepancy was seen in terms of thymus specificity (Fig. 4A), which accounted for the negative correlation. Interestingly, this thymus discrepancy is supported by microarray data [32], which show significantly upregulated expression of *5830405N20Rik *(Fig. 4C), but not of *KIAA0748 *(Fig. 4B). The second most strongly negatively correlated ortholog pair was human *MS4A1 *and its mouse counterpart, *Ms4a1 *(r = -0.96), both of which were preferentially expressed, though to different extents, in B cells, lymphocytes, and leukocytes (Fig. 5A), these preferred tissues were in agreement with reported expression results [33] and with microarray data [32]. The main discrepancy was in the blood vessels, for which mouse *Ms4a1 *exhibited a much higher specificity (*p *= 2.12E-30) than human *MS4A1 *(*p *= 6.42E-02) (Fig. 5A). This discrepancy could not be checked by microarray data, as the blood vessel was not examined in the microarray experiments [32], nor could we find any previous reports on the expression of these two genes in blood vessels. The third most strongly negatively correlated pair was human *SLC2A6 *and mouse *Slc2a6 *(r = -0.87), both of which were preferentially expressed in macrophages, but their brain specificity differed significantly (Fig. 5B). In this case, the data from the two reports on expression of human *SLC2A6 *in brain [34,35] are contradictory and thus could not be used to assess the EST results.

In addition to those expressed in at least three common tissues, 324 orthologs were not expressed in any tissue in common in human and mouse; of these 240 showed a high specificity (*p *< 1E-6) for at least one tissue. One example is that human *HATH6 *was preferentially expressed in stomach ascites (*p *= 2.43E-11) and the stomach (*p *= 5.61E-07), whereas its mouse ortholog, *Atoh8*, was testis-specific (*p *= 2.75E-08). Our literature search revealed one study on *Atoh8*, which indicated that it is a distant mammalian homologue of the Drosophila proneural gene atonal and is expressed in neural cells, as shown by Northern blots, but, in this study, only brain and whole embryo were profiled and no data were given for expression in the stomach or testis [36].

Another human gene, *LY64*, was identified as preferentially expressed in human B cells (*p *= 5.34E-15), leukocytes (*p *= 5.28E-13), lymphocytes (*p *= 1.13E-13), lymph (*p *= 2.45E-10), and whole blood (*p *= 2.16E-12), whereas the mouse ortholog *Ly64 *was highly specific for the colon (*p *< 1E-99) and cecum (*p *= 1.20E-11). This drastic discrepancy was also seen in the microarray data [32]. Mouse *Ly64 *was initially identified as the ortholog of human *LY64 *with 74% amino acid identity [37]. However, another human gene, *MUC13*, was later shown to have 52% amino acid identity to mouse *Ly64*, and both *MUC13 *and *Ly64 *were found to be expressed at highest levels in the large intestine and rectum [38]. In agreement with this, our analysis showed that *MUC13 *was specifically expressed in the colon (*p *< 1E-99).

### Database and website

We have created a web-based database, named HMDEG (a database for Human and Mouse Differentially Expressed Genes), along with search utilities to facilitate free access to, and easy searching of, our results for both normal and diseased tissues. For example, by selecting a specific tissue or organ in the pull-down menu, a full list of genes expressed differentially in that tissue/organ in order of increasing *p *value, along with the corresponding UniGene cluster ID, gene name, and gene description, is displayed. Other search options, such as gene name, EST accession number, and the expression profiles of the corresponding human or mouse orthologs, are also allowed. The whole database is available for download upon request.

## Discussion

**Figure 6:**
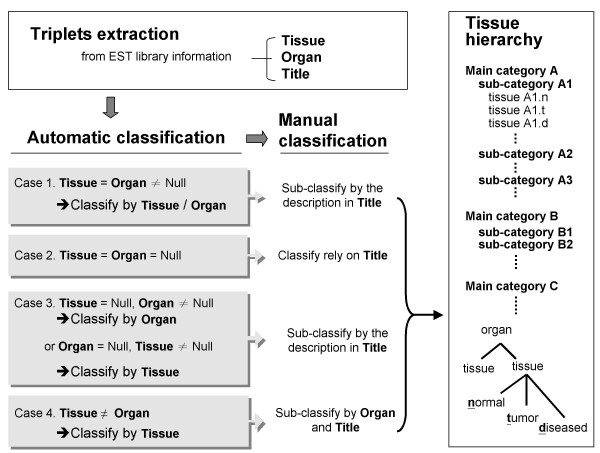
**Library classification procedure**. A triplet of fields consisting of tissue, organ, and title for each EST library from the dbEST report files was extracted. For triplets with records containing the same description under both tissue and organ or no record for one, but not both, the library can be automatically classified into the category described in the tissue/organ field. This automatic assignment was then checked with title to avoid omission of useful information, such as disease state, that could be hidden in the title fields. In some cases (case 2 and 4), the tissue and organ fields were different or both were empty. If different, the library was temporarily classified according to tissue, then modified manually if the other two fields contained more information that could be used for the modification. If both were null, the classification relied on the title.

Knowledge of the tissue in which a gene is specifically or preferentially expressed is often an important clue to its function. The very large database of ESTs has been a useful source for extracting such information by bioinformatics approaches. Several related bioinformatics tools, including the NCBI's Digital Differential Display (DDD) [39], are available, but they usually require the user to manually specify which libraries for the two groups of tissues should be included in the comparison. Others, such as TissueInfo [17] and ExQuest [18], like the present approach, use tissue hierarchies to extract ESTs from the tissue being searched. TissueInfo only includes normal tissues and does not provide quantified expression profiles. Although ExQuest distinguishes between tumor-related and normal tissues, it also does not give quantitative gene expression results [40]. Although more and more EST-based differential expression analyses are being reported, they have so far mostly been confined to specific tissues (e.g. placenta [29], heart [41], and retina [42]). Thus, a convenient and integrated web database that allows users to conduct a large-scale analysis is needed. The present work addressed this need by carefully classifying the EST libraries and creating a database that will allow the user to access the whole statistical test results using search options of both gene and tissue. The procedure used to group EST libraries into tissues (Fig. 6), a task difficult to automate because of different nomenclatures, spelling errors, and other deficiencies in the EST report files, can serve as a template for the cataloguing of new libraries.

The total numbers of genes we tested from normal tissues were 72865 for human and 30172 for mouse. The number of genes classified as "differentially expressed" was dictated by the *p *value threshold (Fig. 1), where one expects more false positives for larger *p *values. The number of false positives, genes falsely classified as "differentially expressed", can be estimated based on Bonferroni correction [43]: at 1E-6 *p *value, for example, the predicted false positives were 0.07 for human (72865 × 1E-6) and 0.03 for mouse (30172 × 1E-6). This and the observation that most genes expressed in 3 tissues or less at *p *< 1E-6 (Table 1) suggested that 1E-6 was a reasonable threshold to use for detecting differentially expressed genes in our analysis. Note also that the *p *value was used here merely as an index to rank expression level and should not be taken as a bona fide probability measure [41].

Overall, our analysis showed that genes identified as differentially expressed by EST analysis generally did not correspond well to those detected by microarray; a similar observation of a weak correlation between the two systems has been previously noted [24]. Nevertheless, as the *p *value threshold of the A-C test defining differential expression became more stringent, the correlation became more evident, although the degree to which this occurred varied with tissue type (Fig. 2). The factors responsible for the discrepancies between different experimental methods and between different tissues remain poorly understood and require future investigations.

Similar to the comparison with microarray, the tissue-based *p v*alue correlation between human and mouse orthologs also became stronger as the threshold for defining tissue-specific orthologs was set smaller, suggesting that tissue-specific orthologs tend to have more similar expression patterns than those lacking significant specificity (Table 2). At *p *< 1E-6, the results of our analysis of a few genes known to be tissue-specific agreed with the published data, and the majority (~60%) of human and mouse orthologs exhibited strong (0.8>r ≥ 0.6) or very strong (r ≥ 0.8) correlations in terms of their tissue distribution and specificity (Table 3).

Orthologs with significant disparity were also observed. Some, such as *KIAA0748*,*MS4A1*, and *SLC2A6*, differed from their orthologous counterpart only in the level of specificity (*p *value). Others, such as *HATH6 *and its mouse ortholog, are preferentially expressed in entirely different tissue(s). Many factors, such as heterogeneity of the tissue samples used to construct EST libraries and insufficient ESTs for theses genes, could contribute to these significant disparities. Inaccurate ortholog pairing is also a potential source of error. For example, with the identification of *MUC13*, it is now evident that *Ly64 *had been mistaken for the ortholog of *LY64*. This mistake has been corrected in a recent release of HomoloGene (on Mar 24, 2005), but is still present in MGI (Mouse Genome Informatics [44]), another widely used curated database of human and mouse orthologous genes. Of course, the observed disparities, especially those substantiated by other sources of data, may indeed represent real phenomena, suggesting that some orthologous genes, despite sharing similar genotypic features, could have disparate phenotypes.

## Conclusion

The present analysis has yielded a useful tool to aid transcriptomic research into human and mouse genes. Obvious applications include the ready retrieval of information on genes expressed differentially in a tissue of interest and the tissue distribution and expression specificity of a particular gene or of a human and mouse ortholog pair. The presence of orthologs with divergent expression profiles may hint at novel functions, divergent regulation, or new ortholog relationship and guide future studies.

## Methods

### Data retrieving, screening, and classifying

Raw data of EST reports from dbEST (at 2003/05/23 for human and 2003/07/10 for mouse) and cluster information from UniGene (build #161 for human and build #128 for mouse) were downloaded from NCBI. We parsed the EST reports to extract EST data of *Homo sapiens *and *Mus musculus*, from which we retrieved the EST unique identifier (GI number), GenBank accession number, and library information, including "Organism", "dbEST lib id", "Lib Name", "Tissue type", and "Organ". For each EST record, we retrieved its corresponding UniGene data, including cluster ID, gene name (gene symbol), and gene description.

For each EST library, we extracted a triplet consisting of title, tissue, and organ from, respectively, the fields "Lib Name", "Tissue Type", and "Organ" in the dbEST report files. Based on the triplet, each library was classified into a corresponding tissue category, according to the TissuDB tissue hierarchy [45]. Our library classification process is illustrated in Fig. 6. Libraries without a definite pathological description in the triplet were considered to be derived from normal tissues. To mitigate variation due to unspecified tissue and artificially modified expression, libraries described as pooled, mixed, subtracted, differentially displayed, normalized, or coming from multiple tissues were excluded. Libraries without a clear description in the triplet were also discarded. There remains the possibility of some artificially modified libraries escaping from this screening, but their effect on the present analysis should be minimized, not to mention that some of them may in fact equalize the expression count, thus making detection of differential expression more stringent.

In all, we downloaded 5,372,149 human ESTs from 8,145 EST libraries and the screening process described above left us with 6,247 libraries and 3,352,546 ESTs distributed in 96,444 UniGene clusters for analysis. Similarly for mouse, 841 EST libraries were downloaded, of which 630 survived the same elimination process, leaving 3,009,721 ESTs (out of 3,132,883) distributed in 30,172 UniGene clusters for analysis.

The 6,247 human libraries were classified by the process shown in Fig. 6 into 157 tissue/organ categories, of which 94 were normal, 53 tumor-related, and 10 related to other diseases. The 630 mouse libraries were classified into 108 tissue/organ categories, of which 99 were normal, 9 tumor-related, and none were related to other diseases. To simplify matters, only the analysis results for normal tissues are presented here; those for diseased tissues will be reported elsewhere.

### A-C test for differentially expressed genes

To profile the genes expressed in a tissue, we extracted the UniGene cluster ID of the ESTs that were classified to the target tissue. For each gene in the target tissue, we performed the A-C test [21] to evaluate tissue specificity:

p(y/x)=(N2N1)y(x+y)!x!y!(1+N2N1)(x+y+1)
 MathType@MTEF@5@5@+=feaafiart1ev1aaatCvAUfKttLearuWrP9MDH5MBPbIqV92AaeXatLxBI9gBaebbnrfifHhDYfgasaacH8akY=wiFfYdH8Gipec8Eeeu0xXdbba9frFj0=OqFfea0dXdd9vqai=hGuQ8kuc9pgc9s8qqaq=dirpe0xb9q8qiLsFr0=vr0=vr0dc8meaabaqaciaacaGaaeqabaqabeGadaaakeaacqWGWbaCcqGGOaakcqWG5bqEcqGGVaWlcqWG4baEcqGGPaqkcqGH9aqpdaqadaqaamaalaaabaGaemOta40aaSbaaSqaaiabbkdaYaqabaaakeaacqWGobGtdaWgaaWcbaGaeeymaedabeaaaaaakiaawIcacaGLPaaadaahaaWcbeqaaiabdMha5baakmaalaaabaGaeiikaGIaemiEaGNaey4kaSIaemyEaKNaeiykaKIaeiyiaecabaGaemiEaGNaeiyiaeIaemyEaKNaeiyiaeYaaeWaaeaacqaIXaqmcqGHRaWkdaWcaaqaaiabd6eaonaaBaaaleaacqaIYaGmaeqaaaGcbaGaemOta40aaSbaaSqaaiabigdaXaqabaaaaaGccaGLOaGaayzkaaWaaWbaaSqabeaacqGGOaakcqWG4baEcqGHRaWkcqWG5bqEcqGHRaWkcqaIXaqmcqGGPaqkaaaaaaaa@56F0@

pvalue(x,y)=∑X=x∞p(y/X)
 MathType@MTEF@5@5@+=feaafiart1ev1aaatCvAUfKttLearuWrP9MDH5MBPbIqV92AaeXatLxBI9gBaebbnrfifHhDYfgasaacH8akY=wiFfYdH8Gipec8Eeeu0xXdbba9frFj0=OqFfea0dXdd9vqai=hGuQ8kuc9pgc9s8qqaq=dirpe0xb9q8qiLsFr0=vr0=vr0dc8meaabaqaciaacaGaaeqabaqabeGadaaakeaacqWGWbaCcqWG2bGDcqWGHbqycqWGSbaBcqWG1bqDcqWGLbqzcqGGOaakcqWG4baEcqGGSaalcqWG5bqEcqGGPaqkcqGH9aqpdaaeWbqaaiabdchaWjabcIcaOiabdMha5jabc+caViabdIfayjabcMcaPaWcbaGaemiwaGLaeyypa0JaemiEaGhabaGaeyOhIukaniabggHiLdaaaa@49A8@

where x and y are the numbers of ESTs clustered in the same gene, but expressed, respectively, in the target tissue and in all other tissues, and N_1 _and N_2 _are, respectively, the total number of ESTs from the target tissue and from all other tissues. Following the criteria for using the Poisson distribution [21], tissues with insufficient ESTs (N_1 _or N_2 _< 1000) and clusters with a biased data set (x ≥ N_1 _× 5% or y ≥ N_2 _× 5%) were excluded from the statistical test.

### Orthologous gene data retrieval and correlation analysis

The raw data of HomoloGene (released on Feb. 2, 2004) were downloaded from NCBI. Using the taxonomy ID of this database, we extracted curated human and mouse orthologous gene pairs and discarded those annotated as putative. For the curated orthologous gene pairs, we obtained their gene names and UniGene cluster IDs and linked them to the expression profiles we had computed using the A-C test. For each ortholog pair expressed in at least 3 tissues in both human and mouse, the association between their expression profiles was analyzed by applying Pearson's correlation to their tissue specificity *p *values. We classified the strength of association, using the absolute value of Pearson's correlation coefficient (r), as follows: 0–0.19 was regarded as very weak, 0.2–0.39 as weak, 0.40–0.59 as moderate, 0.6–0.79 as strong, and 0.8–1 as very strong.

## Authors' contributions

Sheng-Ying Pao conceived the study, participated in its design, carried out the acquisition, analysis, and interpretation of data, coordinated the construction of the database and website, and drafted the first version of the manuscript. Win-Li Lin participated in the design and coordination of the study. Ming-Jing Hwang supervised and participated in all phases of the study. All authors read and approved the final manuscript.

## Figures and Tables

**Table 1:** Percentage of genes identified as differentially expressed, as defined by different *p *values.

A. Human differentially expressed genes					
No. of tissues expressing the gene	5.00E-02	5.00E-03	5.00E-04	5.00E-05	1.00E-06

1	35.76%	41.77%	53.27%	63.02%	62.22%
2	15.19%	20.21%	22.66%	18.73%	20.77%
3	9.65%	12.11%	9.82%	7.66%	6.98%
4	8.69%	10.33%	7.05%	5.56%	5.23%
5	4.97%	5.32%	3.17%	2.21%	2.04%
>5	25.75%	10.27%	4.04%	2.82%	2.75%

B. Mouse differentially expressed genes					

No. of tissues expressing the gene	5.00E-02	5.00E-03	5.00E-04	5.00E-05	1.00E-06
1	27.49%	29.72%	39.33%	47.28%	54.58%
2	8.57%	16.48%	20.04%	21.17%	19.94%
3	10.84%	10.73%	12.97%	12.12%	11.34%
4	7.23%	9.03%	9.52%	7.69%	5.98%
5	4.57%	7.73%	6.21%	4.43%	3.36%
>5	41.29%	26.32%	11.93%	7.32%	4.79%

**Table 2:** Correlation analysis of human and mouse orthologous genes. r indicates the Pearson correlation coefficient of the A-C test *p *values for orthologs expressed in at least 3 normal tissues in common in human and mouse and expressed in at least one human tissue and one mouse tissue with *p *< threshold.

*p *value threshold	No. of ortholog pairs	ave r	ave r (r ≥ 0)	ave r (r < 0)	No. of pairs with r ≥ 0	No. of pairs with r < 0
No	6585	0.20	0.39	-0.25	4571 (69.42%)	2014 (30.58%)
5.00E-02	5199	0.23	0.43	-0.26	3591 (69.07%)	1608 (30.93%)
5.00E-03	2736	0.38	0.50	-0.20	2262 (82.68%)	474 (17.32%)
5.00E-04	1889	0.47	0.56	-0.21	1687 (89.31%)	202 (10.69%)
5.00E-05	1114	0.55	0.61	-0.22	1012 (90.84%)	102 (9.16%)
1.00E-06	892	0.60	0.65	-0.19	836 (93.72%)	56 (6.28%)

ave = average

**Table 3:** Number of significant tissue-specific (*p *< 1E-6) ortholog pairs with different strengths of association. r indicates the Pearson correlation coefficient for the A-C test *p *values for orthologs expressed in at least 3 normal tissues in common in human and mouse and expressed in at least one human tissue and one mouse tissue with *p *< 1E-6.

Strength of association	Number of pairs	Number of pairs with a positive correlation (*r*>0)	Number of pairs with a negative correlation (*r*<0)
0<|*r*|<0.2	138 (15.47%)	99 (11.10%)	39 (4.37%)
0.2≤|*r*|<0.4	98 (10.99%)	91 (10.20%)	7 (0.79%)
0.4≤|*r*|<0.6	136 (15.24%)	130 (14.57%)	6 (0.67%)
0.6≤|*r*|<0.8	160 (17.94%)	159 (17.83%)	1 (0.11%)
0.8≤|*r*|≤1	360 (40.36%)	357 (40.02%)	3 (0.34%)

Sum	892	836 (93.72%)	56 (6.28%)
